# Ovarian endometrioma – a possible finding in adolescent girls and young women: a mini-review

**DOI:** 10.1186/s13048-019-0582-5

**Published:** 2019-11-07

**Authors:** Krzysztof Gałczyński, Maciej Jóźwik, Dorota Lewkowicz, Anna Semczuk-Sikora, Andrzej Semczuk

**Affiliations:** 1 Siedlce University of Natural Sciences and Humanities, Faculty of Natural Sciences, Konarskiego str. 2, 08-110 Siedlce, Poland; 2 Second Department of Gynecological Oncology, St. John’s of Dukla Cancer Center of Lublin, Jaczewskiego str. 7, 20-090 Lublin, Poland; 3 Department of Gynecology and Gynecologic Oncology, Białystok Medical University, Kilińskiego str. 1, 15-089 Białystok, Poland; 4 Department of Clinical Pathology, Lublin Medical University, Jaczewskiego str. 8b, 20-090 Lublin, Poland; 5 Department of Pathology of Pregnancy, Lublin Medical University, Staszica str. 16, 20-081 Lublin, Poland; 6 IIND Department of Gynecology, Lublin Medical University, Jaczewskiego str. 8, 20-954 Lublin, Poland

**Keywords:** Endometrioma, Endometriosis, Ovarian cyst, Adolescence, Adolescen

## Abstract

Young girls before menarche or menstruating adolescent women may experience long-term drug-resistant chronic pelvic pain, as well as other symptoms associated with pelvic mass. In such cases, it is of great importance to consider ovarian endometrioma in the differential diagnosis. In general, endometrioma is recognized as an ovarian cyst. However, in most cases, the pathology represents pseudocyst with a partial or complete endometrial-like lining with extraovarian adhesions and endometriotic implants which are likely to occur at the sites of ovarian adhesions and at the ceiling of the ovarian fossa. Ovarian endometriomas occur in 17–44% patients with endometriosis and account for 35% of all benign ovarian cysts. The time span from the onset of menarche to the time of endometrioma formation, which requires surgical intervention, has been evaluated to be a minimum of 4 years. The pathogenesis of early-life endometrioma may be different from other types of endometriosis. Diagnosis is often delayed, especially in adolescents, who tend to wait too long before seeking professional help. The three specific aims of treatment in adolescents with endometriosis and endometriomas are control of symptoms, prevention of further progression of the disease as well as preservation of fertility. Increasing evidence demonstrates association between ovarian endometriosis and ovarian cancer. In the present mini-review, we draw the particular attention of clinicians to such a possibility, even if relatively infrequently reported.

## Introduction

Menstruating adolescent women or even young girls before menarche may experience long-term drug-resistant chronic pelvic pain, as well as other symptoms associated with pelvic mass found on ultrasound (US), computed tomography (CT), or magnetic resonance imaging (MRI) [1]. In such cases, it is of great importance to consider endometriosis and its local manifestation, ovarian endometrioma, in the differential diagnosis. In the present mini-review, we draw attention of clinicians to such a possibility, even if relatively infrequent.

### Endometriosis

Endometriosis is defined as the presence of endometrial glands and stroma outside the uterine cavity [2]. The implantation of endometrial tissue in the peritoneal cavity through retrograde menstruation is the most accepted theory of endometriosis but its etiology is still poorly understood [3]. A reflux of endometrial cells into the abdominal cavity during menstrual bleedings is a normal condition which occurs in 90% of menstruating women with patent fallopian tubes, although the disease develops only in subjects with hormonal or immune disorders [4, 5]. In women affected by endometriosis, the peritoneal fluid contains: elevated levels of immune cells which demonstrate increased susceptibility to apoptosis, elevated concentrations of pro-inflammatory mediators/cytokines such as tumor necrosis factor-α, interleukin-1β, and interleukin-6, dysfunctional macrophages and NK cells, and highly accumulated regulatory T suppressor cells which promote inflammation as well as stand behind the initiation and progression of endometriosis-associated ovarian cancer [6–8]. Endometriosis is associated with alteration in hypothalamus-hypophysis-ovary axis leading to changes in the concentration of estradiol, progesterone, luteinizing hormone (LH), and follicle-stimulating hormone (FSH) in the serum, peritoneal fluid and follicular fluid of women with endometriosis. The ectopic endometrium presents persistent estrogen receptors (ER) hormonally independent during the luteal phase. In addition, endometriotic implants express aromatase which catalyzes conversion of androgens to estrogens suggesting that local estrogens production can increase estrogen concentration and together with circulating estrogen can stimulate the growth of endometriotic lesions. The action of progesterone mediated via progesteron receptor (PR) is also altered in endometriotic patients. The PROGINS polymorphism of PR decreases the stability of receptor which loses its capacity to inhibit the activation of the ER and thus exposing endometrium to greater action of estrogens. Increased level of LH was observed in the peritoneal fluid of infertile women with endometriosis. Additionally, endometriotic patients have a lower concentration of LH receptor (LHR) in corpus luteum and follicles during the early and late follicular and late luteal phase compared to healthy controls. In severe endometriosis LHR concentration is extremely low [9]. Current data suggest that also FSH action mediated by FSH receptor (FSHR) is disrupted in endometriotic patients due to changes in signaling pathways [10]. Usually, peritoneal, ovarian and rectovaginal endometriosis are distinguished based on the most frequent localization [11]. In the general population of women of reproductive age, the incidence of endometriosis is estimated to be approximately 15%, however, in groups of patients with chronic pelvic pain and infertility, up to 60 and 50% of them may suffer from it, respectively [12–14].

Endometriosis is described as premenarcheal and distinguished from adolescent when lesions and associated symptoms occur before menarche, mainly during thelarche [4]. It is rather difficult to establish the prevalence rate of endometriosis among adolescents [15]. The symptoms often start at young age. Interestingly, the mean duration between their onset and final diagnosis is 22.8 months and the mean number of physicians who have seen the patient before the diagnosis is reached is 3 [14, 16]. Some studies report that due to misinterpretation of clinical symptoms the diagnosis may be delayed even by 8–10 years [17]. Girls and women who see a gynecologist first for symptoms related to endometriosis are more likely to report a shorter time to diagnosis, see fewer physicians, and report a better experience overall with their physicians during their diagnostic experience, probably because gynecologists are more familiar with symptoms of endometriosis than other physicians [12]. About two thirds of adult women with endometriosis report symptoms arising before 20 years of age [18]. Patients with endometriosis mainly report symptoms of pain (including chronic pelvic pain), dysmenorrhea and, if sexually active, dyspareunia [19–21]. About two-thirds of adolescent girls with chronic pelvic pain or dysmenorrhea have laparoscopic evidence of endometriosis. About one-third of these adolescents with endometriosis have moderate-severe disease [22]. Data from a retrospective analysis of adolescents with histologically confirmed endometriosis showed that the most common complaints were dysmenorrhea (64%), menorrhagia (44%), irregular, abnormal uterine bleeding (60%) and at least one genito-urinary symptom (52%) [23]. Dysmenorrhea is likely to be a precursor in the disease development and shorter/shortened cycles may possibly suggest the increased risk [24]. Adolescents with endometriosis are more likely to experience migraines than those without endometriosis [25]. In epidemiologic studies, several early-life factors were identified which include prenatal exposure to diethylstilbestrol and cigarette smoking, and altered hormonal milieu and exposure to regular soy formula feeding during infancy [20]. Some other data indicate that decreased abilities of women to contribute to the society because of the disease amount to the economic burden of 22 billion in the United States. A delay in the diagnosis escalates the economic impact of endometriosis, especially in adolescents who tend to wait too long before seeking professional help [17]. Early diagnosis and treatment of endometriosis seem to be crucial for young patients, increases their quality of life, brings relief of symptoms and decreases morbidity and negative impact of the disease on future fertility. Studies show that the longer the diagnosis is delayed, the more the endometriosis is in an advanced stage at the time of laparoscopy. Treatment in early-stage endometrioma provides less damage to the ovary by a less invasive surgical procedure which decreases the risk of iatrogenic premature ovarian failure. Long-term ovarian endometriosis leads to persistent inflammation resulting in fibrosis of the ovarian cortex and loss of follicles and smooth muscle cell metaplasia [4].

Girls before menarche may experience drug-resistant chronic pelvic pain, therefore it is of great importance to include endometriosis in the protocols of differential diagnosis. Pain may remain untreated for a long time, even above 6 months, and may interfere with the patient’s daily activities [1]. A possible origin of symptoms from the gastrointestinal, genitourinary and musculoskeletal systems and psychosocial aspects should also be taken into consideration [26]. Another problem may arise from the presence of Müllerian duct anomalies (such as unicornuate uterus with a non-communicating rudimentary horn or uterus didelphys with a vaginal septum) when, due to secondary obstruction in vaginal menstruation, retrograde tubal menstruation and transportation of endometriotic implants into the abdominal cavity are enhanced [27, 28]. A very early presentation of endometriosis should prompt consideration of Müllerian anomaly with outflow obstruction [29].

Endometriosis is staged according to the revised classification of the American Society for Reproductive Medicine and determined on the basis of size, location, and type of lesion(s) and the extent of adhesions [23, 30]. The clinical manifestation of endometriosis varies between adolescents and adults. Young patients report severe primary dysmenorrhea which is often resistant to non-steroidal antiinflammatory drugs and oral contraceptives. The appearance of peritoneal lesions is also different. In adolescents, endometriotic implants are florid (clear or red papules, vesicular implants) with minimal fibrosis. In contrast, in adult patients, black implants with dense fibrotic tissue are common findings [5, 23]. Obstructive genital tract anomalies often accompany adolescent endometriosis whereas in adults rectal and bladder endometriosis and uterine adenomyosis are concomitant pathologies [16]. In a recent study, Harris and co-workers reported an increased risk for endometriosis in patients who experienced early-life sexual or physical abuse [31].

### Early onset endometriosis (EOE)

Endometriosis in adolescent patients may have a different origin from that seen in adult women. A potential cause involved in the development of EOE is neonatal uterine bleeding (NUB) which leads to the seeding of endometrial progenitor cells into the pelvic cavity which become activated around thelarche. These dislocated cells implanted on the pelvic organs remain dormant for years and are activated in the presence of factors leading to the development of highly angiogenic implants, recurrent ectopic bleeding, and the formation of endometriomas which seems to be a characteristic feature of this type of endometriosis [16]. NUB occurs in approximately 5% of female neonates as an endometrial response to progesterone. The bleeding itself is an effect of progesterone withdrawal. In two-thirds of neonates, the endometrium is proliferative and resistant to progesterone. This resistance persists till menarche, and during first years of adolescence. Occurrence of fetal distress caused by preeclampsia, fetal growth restriction, post-maturity as well as Rhesus isoimmunization is significantly associated with NUB. These feto-maternal factors characterized by insufficient blood supply of placenta and fetal hypoxia promote decidualization of fetal endometrium and sensitizes it to progesterone [32]. In the pelvis, endometrial cells and stroma attach quickly to the peritoneum [5, 16]. This theory convincingly explains why endometriosis and endometriomas can occur in girls before their first menstruation and why adolescents can suffer from advanced endometriosis. Benagiano et al. underline that EOE can become hidden, debilitating and progressive disease that impairs the patient’s future reproductive life [16].

### Endometrioma

The most common sites of endometriosis are the ovaries, followed by the Douglas pouch, the posterior leafs of the broad ligaments, and the sacrouterine ligaments [33]. Ovarian endometriomas occur in 17–44% patients with endometriosis and account for 35% of all benign ovarian cysts. The time span from the onset of menarche to the time of endometrioma formation which requires surgical intervention has been evaluated to be a minimum of 4 years [2, 15]. The pathogenesis of early-life endometrioma may be different from other types of endometriosis such as peritoneal implants and rectovaginal nodules [34]. There are a number of theories explaining the development of endometriotic cysts [4]. Endometrioma may be formed due to inversion and subsequent progressive invagination of ovarian cortex with endometriotic implants which fill the cyst with hemolyzed blood. Another explanation is metaplasia of invaginated ovarian celomic epithelium which creates active endometrial tissue. Also, ovarian follicular fluid may potentially induce endometrial cell growth [2]. Active endometrial cells implanted on the surface of the ovary secrete matrix metalloproteinases which can lyse the extracellular matrix thus allowing the ectopic cells to infiltrate the ovary and leading to the destruction of the healthy tissue. This up-regulation of matrix metalloproteinases is mediated by tenascin, which modify cell adhesion. Additionally, follistatin and urocortin are overexpressed in endometriomas, concentrations of which are elevated in the serum. Moreover, high concentrations of inhibin A, inhibin B, and activin A in follicular fluid may stimulate growth and differentiation of endometriotic cells. Epithelial and stromal endometriotic cells vary from normal endometrium at a molecular level. Different expression of more than 100 genes was found in ectopic endometrium compared to eutopic one. These alterations are associated with cell adhesion, inflammation and remodeling of extracellular matrix. Progesterone resistance, increased estrogen receptor activity, local estrogen production via aromatase activity are caused by genetic and epigenetic changes which are a result of disrupted estrogen–progesterone receptor expression [35]. Interestingly, endometriomas are more common in the left than in the right ovary [36, 37]. For example, in the group of 206 patients with endometrial cysts, Matalliotakis and *co-workers* found endometriomas located twice more frequently in the left ovary (67.4%) than in the right one (32.6%). These authors suggested that the presence and growth of endometriomas are related to anatomic variables, namely anatomic asymmetry (decreased fluid movement on the left side due to the nearby presence of the sigmoid colon and the left broad ligament). Indeed, the compression syndrome of left renal vein due to the incompetent and dilated left ovarian vein leads to venous congestion and the resultant hypoxia and increased concentrations of sex hormones and cytokines which may explain this phenomenon [37].

**Fig. 1:**
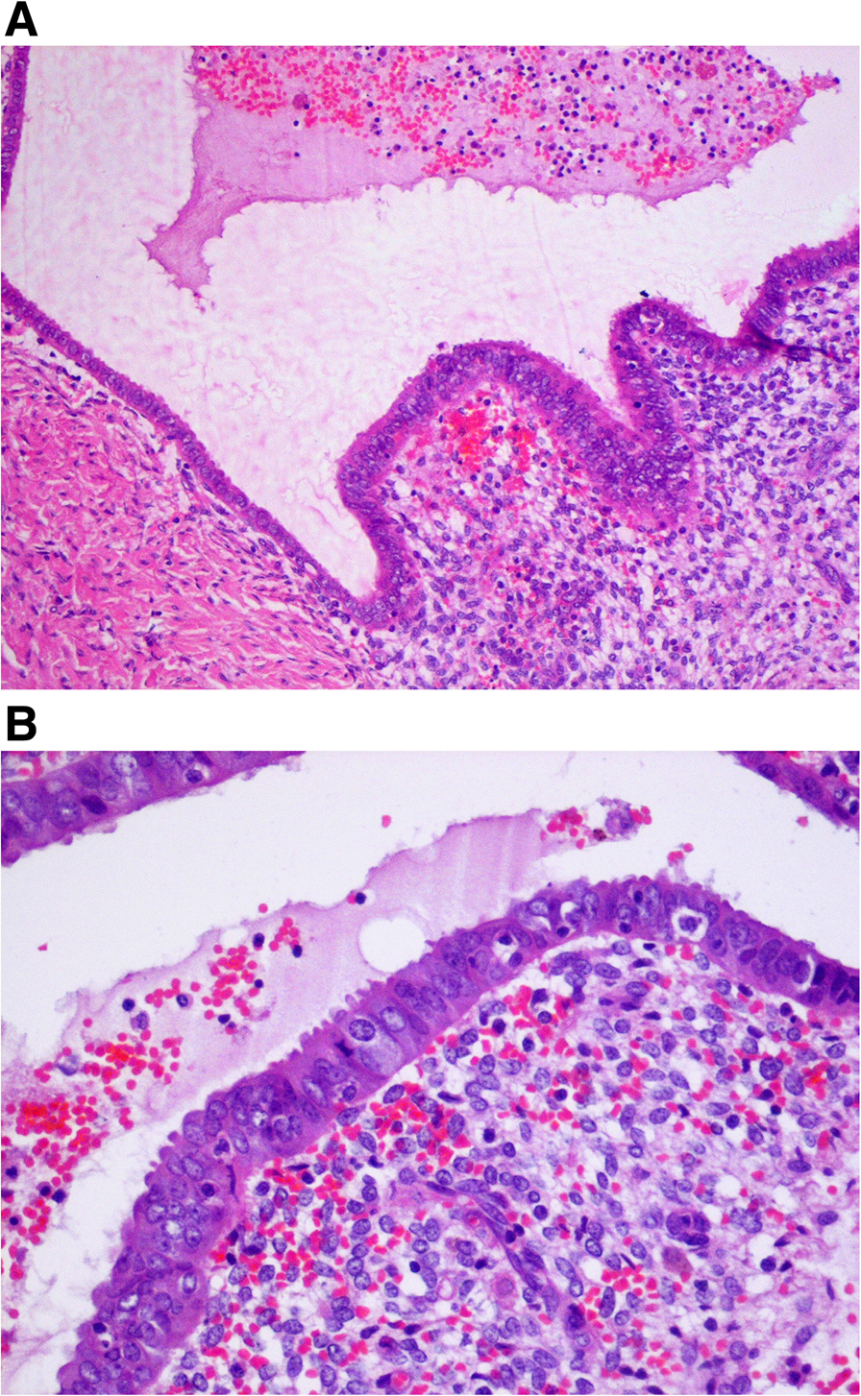
**a**, **b** Typical histopathological images of the wall of endometrioma – sample collected during laparoscopic cyst enucleation at a 20-years-old woman (100x and 200x magnification, respectively)

Four different types of endometriomas can be distinguished: cortical invagination cysts, surface inclusion cyst-related endometriotic cysts, physiological cyst-related endometriotic cysts, and unclassified type. The presence of oocytes in the inner wall of the cyst is a proof of an inner cortex inclusion and allows the diagnosis of the cortical invagination type. However, this finding depends on patient age and may be influenced by fibrosis, smooth muscle metaplasia, as well as stretching of the cortex [38]. In general, endometrioma is recognized as an ovarian cyst. However, in most cases, the pathology represents pseudocyst with a partial or complete endometrial-like lining with extraovarian adhesions and endometriotic implants which are likely to occur at the site(s) of ovarian adhesions and invagination and at the ceiling of the ovarian fossa [39]. Figures 1 A and B represent typical histopathological images of the wall of endometrioma. In some studies, the presence of endometrioma was associated with adhesions to the posterior leaf of the broad ligament in as many as 98% of the cases, and these adhesions were classified more frequently as deep (70.5% of cases) than superficial (29.5%) [38, 40]. Hydroureter and hydronephrosis secondary to a pelvic mass may be present in patients with large endometrioma [2].

The evaluation of ovarian cortical tissue from women with endometrioma revealed a reduced volume of normal ovarian tissue in the distended ovarian cortex, a finding not observed to this degree in other benign cysts. In comparison with the healthy organs, ovaries with endometrial cysts have a reduced responsiveness after exogenous gonadotropin stimulation, lower antral follicles count, lower follicular density in the cortex, increased follicular atresia, and increased activation of early follicular development. Furthermore, the density of primordial follicles is reduced and the general morphology and vasculature network are distorted as well. The follicular loss may occur even at early stages of the cyst development. Fibrosis is frequently evidenced in the ovarian cortex derived from endometriomas. The cortex shows increased oxidative stress compared to other benign cysts. Along the growth of many benign ovarian tumors, this cortex becomes stretched and thinned, however, in the presence of endometrioma it additionally contains hemosiderin-laden macrophages and fibrotic components [34, 41, 42].

To date, only a few studies evaluating the clinical characteristics of endometrioma(s) in adolescents have been published (Tab. 1).

**Table 1:** Studies on ovarian endometriomas in adolescents published to date

Authors	Patient age	Presentation	Symptoms	Treatment
Wright and Laufer, 2010 [2]	18	On US and CT: huge pelvic mass of 35 cm in diameter, with solid and cystic components, ascites present. On surgery: large right and left ovarian masses with adhesions to the omentum, pelvic sidewalls, fallopian tubes, and uterus, the combined contents were ~ 8 L of chocolate-brown fluid.	No symptoms, regular menses, no dysmenorrhea, mild hydroureter and hydronephrosis, CA125 = 379.0 U/mL, LDH = 245.0 IU/L.	Laparotomy, enucleation of the cyst in one ovary, drainage of that in the other.
Gogacz et al., 2012 [26]	11	On US, a well encapsulated tumor (capsule approximately 3 mm thick) with homogeneous content, located behind the uterus. On surgery, a left ovarian cyst located in the Douglas pouch, containing chocolate-brown fluid, with numerous adhesions to the peritoneum and intestine.	Premenarcheal vomiting, severe hypogastric pain.	Laparotomy, enucleation of the cyst.
Lee et al., 2013 [19]	Mean age = 19.2 ± 1 ys (*n* = 35)	Bilateral cysts in 49% of cases, located in the right or left ovary in 20 and 31%, respectively. Cul-de-sac obliteration in 57%.	Pain in 77% of cases, incidental in 23% of cases	Laparoscopy, enucleation of the cysts.
Lee et al., 2017 [15]	Mean age = 19.1 ± 1.2 ys (*n* = 105)	Mean cyst size 75 ± 29 mm, bilateral in 21% of cases, located in the right or left ovary in 42.9 and 36.2%, respectively. Complete or partial cul-de-sac obliteration in 14.3 and 32.4%, respectively.	Dysmenorrhea in 40.5% of cases, pelvic pain in 18.8%, gastrointestinal symptoms in 6%, mass effect in 18.8%, incidental detection of endometrioma in 9.4%.	Laparoscopy, enucleation of the cysts.

CA 125 – cancer antigen 125 concentration in serum, LDH – lactate dehydrogenase activity in serum

### Diagnosis

The initial imaging technique for the diagnosis of endometrioma is US examination, which is nowadays widely available, well-accepted, and allows extensive exploration of the pelvis [43]. However, MRI demonstrates an important advantage over other techniques in allowing complete imaging of all pelvic compartments at a time. Also CT can be applied for the diagnosis of endometriosis and revealing various endometriosis-related complications and unusual implantation sites.

Laparoscopy remains the “gold standard” in the final diagnosis of endometriosis and its ovarian manifestation [27, 44]. Almost 50% of adolescents in whom endometriosis is diagnosed at the time of laparoscopy have a severe disease [45]. With enhanced magnification offered by the modern laparoscopic equipment, all endometriotic sites can be identified [46]. Principal features of endometriomas gained from two most popular imaging techniques are presented at Table 2.

**Table 2:** Main features of endometrioma images on US and MRI examinations [27, 44]

Technique	Endometrioma Image	Suspicion of malignant transformation
US	Unilocular or multilocular (less than 5 locules) cysts. Homogenous low-level echogenicity (ground glass echogenicity). Poor or no vascularization. Presence of diffuse low-level echoes. Multilocularity of hyperechoic foci in the wall. Blot clots or fibrin adjacent to the cyst wall forming papillations (no vascularisation inside). Thin septa in large endometriomas.	Anechoic thin-walled cyst with echogenic vegetation or focal wall nodularity (blood clots or fibrosis due to recurrent hemorrhage can mimick these findings).
MRI	Specific sign – shading (caused by old blood products containing high levels of iron and protein). Higher T1, lower T2 signal intensities than in hemorrhagic cysts. Shortening of T1 and T2 secondary to high protein concentration and increased viscosity. Bilateral and multifocal lesions.	Cystic mass containing mural nodules and hemorrhagic fluid. Enhancing mural nodules within endometrioma on T1 W1 is highly suggestive of malignancy. Absence of characteristic T2-weighted “shading” which disappears in malignant tumor.

*US* Ultrasound, *MRI* Magnetic Resonance Imaging

Although endometriosis is closely related to infertility, some women with endometriomas conceive naturally or with the help of assisted reproductive techniques. Therefore, endometrioma is the most common adnexal mass detected during pregnancy [38].

### Treatment

The three specific aims of treatment in adolescents with endometriosis are: control of symptoms, prevention of further progression of the disease as well as preservation of fertility [29, 47]. To minimize pain and disease burden, non-steroidal anti-inflammatory drugs, GnRH agonists, progestins and oral contraception pills are mainstream therapeutic options. Endometriomas do not respond to medical therapy alone, thus usually surgical treatment is necessary. A decision to perform surgery in the adolescent patient can be difficult because of the patient’s fear of surgical intervention and because of potential peri- and post-operative complications [17]. Laparoscopic endometrioma excision is recommended for ovarian cysts larger than 4 cm in diameter [34]. The guidelines of the *European Society of Human Reproduction and Embryology* recommend that endometriomas above 3 cm should be removed before in vitro fertilization (IVF) procedure. Brosens et al. [38] noted that as far as the endometrioma size is concerned, no consensus on a *cut-off* value exists above which surgical treatment should be offered to the patient. One of the most important points is that following ovarian surgery a significant reduction of the ovarian reserve due to the damage to the healthy ovarian tissue may occur. Endometriomas themselves could also be linked to this process. Some authors suggest that surgery, that is, performed at early stages of endometrioma development, may alleviate the local inflammatory environment in the diseased ovaries and thus protect them. They emphasize the role of induced by endometrioma local inflammation which causes “burnout” of early follicles in the ovary. This effect was observed at an early stage of endometrioma formation (1–4 cm in diameter). Active management of small cysts may potentially prevent follicle loss [34, 42]. Endometriomas of 6 cm or more in diameter may be associated with increased risks for infection, rupture, and even malignancy, and therefore, surgical intervention is considered obligatory. The level of expertise in endometriotic surgery is inversely correlated with inadvertent removal of healthy ovarian tissue along with the endometrioma capsule [38]. Due to disruption and disorganization of the cortical wall and loss of identity of the inner cortex, this layer may be difficult to recognize. During cystectomy, identification of cleavage planes becomes difficult leading to unwitting destruction of healthy ovarian tissue [4]. At present, in some centers expectant management is being proposed rather than surgical removal of the cyst. Brosens et al. reported that current management of endometrioma is changing from overtreatment to undertreatment which might be an unfavorable approach because endometrioma is not a simple chocolate cyst which has a tendency to spontaneously disappear, but it is associated with inflammation independent of the lesion’s size, leading to fibrosis of the ovarian cortex, smooth muscle cells metaplasia, and loss of oocytes. These authors concluded that ectopic endometrial tissue should be removed irrespective of the size of the cyst and duration of the disease [38]. Laparoscopic treatment of endometriosis and excision of endometriomas were also associated with improvements in pain relief [48]. Although laparoscopy is traditionally recommended, transvaginal endoscopy is also safe and it is most effective in the treatment of endometriomas that are not larger than 3 cm in diameter [4]. It is worth to mention, taking into consideration future fertility of young women, that surgical treatment of endometrioma undergoing IVF did not alter the outcome of procedure compared to women who did not receive intervention. Women with endometrioma undergoing IVF had similar reproductive outcomes compared with those without the disease, although their cycle cancellation rate is significantly higher [49].

### Recurrence

Lee and *co-investigators* [15] assessed the possibility of recurrence of endometrioma in adolescents after the first-line surgical intervention, where the Kaplan-Meier method was applied. After 24, 36, 60 and 96 months, the cumulative recurrence rates were 6.4, 10, 19.9 and 30.9%, respectively. For adults, the recurrence rate of endometrioma after primary surgery is approximately 12–30% after 2–4 years of follow-up. In the Lee study, diameter of the tumor, stage of endometriosis, coexistence of deep-infiltrating endometriosis, or uni- or bi-lateral involvement of ovaries were all not associated with the risk of recurrence. The median time to recurrence was 53 months. Also, increase in recurrence is associated with age of patients – 4.6% among women aged 20–30 years and 13.2% among women older than 30 years old [15]*.* Some studies recommend postoperative hormonal suppression to reduce risk of recurrence of endometriosis but some authors didn’t support this concept [46].

### Endometrioma and cancer

Increasing evidence demonstrates association between ovarian endometriosis and ovarian cancer [50]. The malignant transformation may occur in up to 1% of endometrioma cases, mostly from ovarian lesions [6]. The risk increases with advanced age at endometrioma diagnosis as well as the size of the lesion. The diameter above 9 cm and post-menopausal age were independent predictive factors for the development of ovarian cancer. Endometriosis-associated ovarian cancer affects women 10–20 years earlier than those suffering from ovarian cancer without endometriosis [27]. In the group of women with endometrioma, 0.72% developed histologically proven ovarian cancer, with clear-cell and endometrioid-type histotypes in 39 and 35% of cases, respectively. Loss of heterozygosity and genomic instability were observed in endometriotic tissue and represented events with highly mutagenic potential. The most common implicated genes are *ARID1A, PTEN, K-ras, p53, HNF,* and the microRNAs. Inactivation of *PTEN* occurs at the beginning of malignant transformation of endometriosis and was found in > 75% of endometriosis-associated ovarian tumors and 15% of endometriotic lesions. Mutations of *p53* are found in approximately 80% of high-grade serous ovarian cancers and are often present in the region of transition atypical endometriotic lesions to ovarian cancer. Mutations of K-*ras* were found in 10–20% of cancers associated with endometriosis. Clear-cell cancer can be preceded with expression of *HNF* in endometriotic lesions. In-activating mutations of chromatin-remodeling *ARID1A* have been found in 49% clear-cell and 30% endometrioid-type ovarian cancers [51]. Some studies suggest other possible mechanisms through which endometriosis can be associated with development of ovarian cancer, such as persistent inflammatory process with cytokines and mediators derived from endometriosis environment, higher levels of estrogens observed in endometriosis and oxidative stress [52]. Consequently, at present there is sufficient evidence to conclude that women with histologically proven endometriosis are at increased risk of developing clear-cell and endometrioid-type ovarian malignancies [53]. Both these histologic types of cancer share similar gene expression pattern, consistent with common origin. Atypical endometriosis, often found in the direct continuity with the tumor, may be a proof of a transition from benign to malignant disease and accompanied 60–80% of all endometriosis-associated ovarian cancers. In atypical lesions, large pleomorphic nuclei, increased nuclear-to-cytoplasmic ratio, cellular crowding, and stratification of tufting were observed [6]. A ruptured ovarian endometrioma can mimick ovarian neoplasm and be a diagnostic dilemma due to high concentration of CA 125 in the serum [54]. Some data suggest correlation between endometriosis and other cancers, such as non-Hodkin lymphoma, breast cancer (BC), melanoma, kidney and endocrine cancers, but this association has not been clarified, yet [52]. Possible pathogenetic pathways that relate endometriosis and breast cancer were assessed in several studies. In a retrospective study, which evaluated the familial risk of BC in women with endometriosis, it was found that 26.7% of women with endometriosis and 5% of women without endometriosis had a positive family history of BC. Reduction in risk of BC in women with bilateral oophorectomy with hysterectomy due to endometriosis was observed in one study. Early interruption of inflammatory process which may contribute to BC may be explanation of this result. Additionally, the treatment of endometriosis could also be implicated in breast carcinogenesis. The hypotesis that endometriosis and BC could share BRCA1 and BRCA2 genes mutations was not confirmed [52, 55].

## Conclusions

Both young and adolescent girls may experience drug-resistant chronic pelvic pain before menarche, therefore, it is of great importance to consider endometriosis in the diagnostic protocol. Endometriosis-like symptoms in girls before the age of 15 years or before menarche indicate the need to screen such girls as early as they develop prominent symptoms. Obstetrician-gynecologists are more familiar with symptoms of endometriosis than other physicians and may be more likely to pursue endometriosis as a diagnostic dilemma, especially in girls with dysmenorrhea and chronic pelvic pain resistant to hormonal treatment.

## Data Availability

Data sharing is not applicable to this article as no datasets were generated or analyzed during the current study.

## Abbreviations

BR: Breast Cancer
CA 125: Cancer Antygen 125
CT: Computed Tomography
EOE: Early Onset Endometriosis
ER: Estrogen Receptor
FSH: Follicle-stimulating Hormone
FSHR: Follicle-stimulating Hormone Receptor
LDH: Lactate Dehydrogenase
LH: Luteinizing Hormone
LHR: Luteinizing Hormone Receptor
MRI: Magnetic Resonance Imaging
NK: Natural Killer Cells
NUB: Neonatal Uterine Bleeding
PR: Progesteron Receptor
US: Ultrasound
